# Vascular Closure Devices after Endovascular Procedures in Swine: A Reliable Method?

**DOI:** 10.1155/2014/514942

**Published:** 2014-03-11

**Authors:** P. Isfort, T. Tanaka, T. Penzkofer, P. Bruners, R. Tolba, C. K. Kuhl, A. H. Mahnken

**Affiliations:** ^1^Department of Diagnostic and Interventional Radiology, RWTH Aachen University Hospital, Pauwelsstrasse 30, 52074 Aachen, Germany; ^2^Department of Radiology, Nara Medical University, 840 Shijo-cho, Kashihra 634-8522, Japan; ^3^Institute of Animal Research, RWTH Aachen University Hospital, Pauwelsstrasse 30, 52074 Aachen, Germany; ^4^Department of Diagnostic and Interventional Radiology, Philipps-University, Baldingerstrasse, 35043 Marburg, Germany

## Abstract

*Purpose*. To investigate the safety and feasibility of the use of a vascular closure device (VCD) after endovascular procedures in swine. *Material and Methods*. In a study on endovascular therapy, VCD (StarClose, Abbott Vascular, Il, USA) was used in 20 female swines to achieve immediate hemostasis after percutaneous right femoral artery (FA) access. 10 animals were sacrificed immediately after the study and 10 animals were sacrificed 28 days after the initial study. To ensure complete hemostasis and patency of the femoral artery, a CT-angiography of the puncture site was performed on day 1 (acute and chronic group) and day 28 (chronic group). After the sacrifice, the femoral artery was explanted and examined macroscopically for signs of VCD dysfunction. *Results*. Technical success rate was 100% with immediate hemostasis being achieved in all animals. No animals showed evidence of hematoma. During explantation, only small traces of coagulated blood were found in the acute group, while there were no signs of hematoma in the chronic group. CT-angiography immediately after VCD application as well as before sacrifice (chronic group) showed patency of the FA in all cases. *Conclusion*. The use of VCD to achieve hemostasis after endovascular studies in swine is feasible and safe.

## 1. Introduction

Vascular closure devices are increasingly used after various types of endovascular procedures including coronary, cerebrovascular, and peripheral vascular interventions as an alternative to manual compression to achieve hemostasis after cannulation of the right femoral artery (FA). Manual compression of the puncture site is associated with prolonged bed rest and a local complication rate (hematoma, bleeding, pseudoaneurysm, and arteriovenous fistula) of up to 9% [1–3]. In contrast, the systematic use of VCD can lower the local complication rate to as low as 0.53% [4]. Nevertheless, the superiority of VCD compared to manual compression with regards to bleeding complications has not been demonstrated in a randomized-controlled trial comparing the methods directly. Minimizing access site complications, patient immobilization, shortening patients' hospital stay, and thereby minimizing the costs of these interventions are the key towards the success of percutaneous endovascular procedures.

In endovascular studies, involving FA cannulation of swine manual compression and prolonged rest is not an option since the animals will move as soon as they awake from anesthesia. Moreover, due to the different anatomical situation in swine with the FA located deeper than in humans, sufficient compression of the punctured vessel against the femoral head is not possible. In this scenario, the use of VCD is indispensable. To date, no information about the safety and efficacy of VCD in swine is available.

The aim of this study is to examine the safety and feasibility of the use of VCD (StarClose) to achieve hemostasis after puncture of the FA.

## 2. Material and Methods

### 2.1. Study Design

In the context of an animal endovascular study, the puncture site in the right FA was closed by means of VCD in *n* = 20 female swine. *N* = 10 animals were sacrificed after the immediate CT-control (acute group) and *n* = 10 animals were sacrificed 28 days after the procedure and after a repeated CT-control (chronic group).

After animals were sacrificed, the punctured groin was carefully dissected in order to evaluate local complications like hematoma or laceration of the FA. In one exemplary case, the right FA was excised in the region of the puncture site for photographic documentation.

### 2.2. Animal Experiments

All experiments were performed in accordance with the German legislation governing animal studies. Official permission was granted from the governmental animal care office (Landesamt für Natur, Umwelt und Verbraucherschutz Nordrhein-Westfalen, Recklinghausen, Germany). Female German landrace pigs from a disease-free barrier breeding facility were housed in ventilated rooms and allowed to acclimatize to their surroundings for a minimum of 5 days before surgery. The animals, weighing around 60 kg, were fasted 12 hrs prior to the experiments. For premedication, the animals received an intramuscular injection of 4 mg kg^−1^ azaperone (Stresnil, Janssen, Germany). Anesthesia was induced by intravenous injection of 3 mg kg^−1^ propofol followed by oral intubation. The animals were ventilated with 40% oxygen at 20–26 bpm and a tidal volume of 10 mL kg^−1^ to keep the end tidal partial carbon dioxide tension (pCO2) between 36 and 42 mmHg. Anesthesia was maintained with isoflurane at a concentration of 1%–1.5% (Forane, Abbott, Germany) and fentanyl (fentanyl, Janssen, Germany) at a concentration of 3-4 mg kg^−1^. To compensate for basic fluid requirements volume, animals received Ringer's lactate (RL) solution at a rate of 4 mL kg^−1^; after laparotomy, the constant infusion rate was set to 8 ml kg^−1^ and not changed until infliction of trauma.

### 2.3. Vascular Closure Device

An FDA-approved VCD (StarClose, Abbott Vascular; Abbott Laboratories, Abbott Park, Il, USA) using a clip-mediated sealing mechanism was used in this study. Therefore, the vascular sheath (5F) in the FA has to be exchanged over a stiff guide wire for the system specific sheath. Thereafter, the tip of the system containing the sealing clip is introduced into the sheath until the system clicks into place. After that, the sheath is split and the sealing clip closes the puncture site. No local compression of the puncture site is performed.

### 2.4. Imaging Follow-Up

After applying the VCD contrast-enhanced Angio-CTs after administration of 123 mL iopromide (Ultravist 300, Bayer Healthcare, Berlin, Germany) using a 64-slice dual-source CT (Siemens SOMATOM Definition, Siemens Healthcare Sector, Erlangen, Germany). Imaging follow-up was performed immediately after the intervention (acute and chronic group) as well as 28 days after the intervention (chronic group). Exemplary CT visualization using volume rendering technique (VRT) was performed using OsiriX (Version 5.6, 64 bit, http://www.osirix-viewer.com/).

## 3. Results

In the control-CTs after the intervention, technical success rate was 100% with no signs of occlusion or extravasation and preserved blood flow in the FA in all animals. Clinical success rate was also 100% with preserved perfusion of the FA in the control-CTs 28 days after vascular closure.

**Figure 1:**
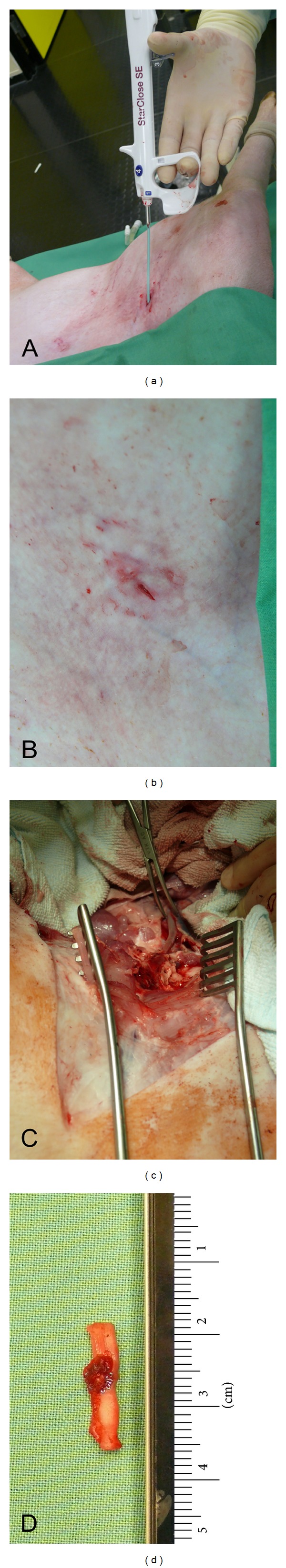
(a) Application of the VCD in pigs right FA. (b) No bleeding after application of the VCD. (c) Only subtle subcutaneous bruising and no significant hematoma visible in dissection of the right groin. (d) Right FA specimen with metallic clip affixed to the vessel wall.

After the animals were sacrificed, no signs of significant complications like significant hematoma surrounding the puncture site or laceration of the FA were observed. The metallic clip closing the puncture site was visibly affixed to the artery wall in all cases. In the acute group, small amounts of blood were visible in the region of the puncture site, which can be considered as normal leakage during the intervention. See also Figure 1 for VCD application and resected artery specimen.

## 4. Discussion

Vascular closure devices (VCD) aim at shortening of haemostasis time and reduction of peripheral vessel complications after arterial puncture. Today, a number of different VCDs are available which can be subclassified into four groups: (1) collagen-based systems (e.g., Angio-Seal, St. Jude Medical, Minnetonka, MN, USA), (2) surface pads with haemostatic substances (e.g., Chito-Seal, Abbott Vascular Devices, CA, USA) as an assisted manual compression, (3) suture-mediated or rather clip closure systems (e.g., StarClose, Abbott Vascular Devices, CA, USA), and (4) multiple-component systems (e.g., DUETT, Vascular Solutions, Minneapolis, MN, USA).

**Figure 2:**
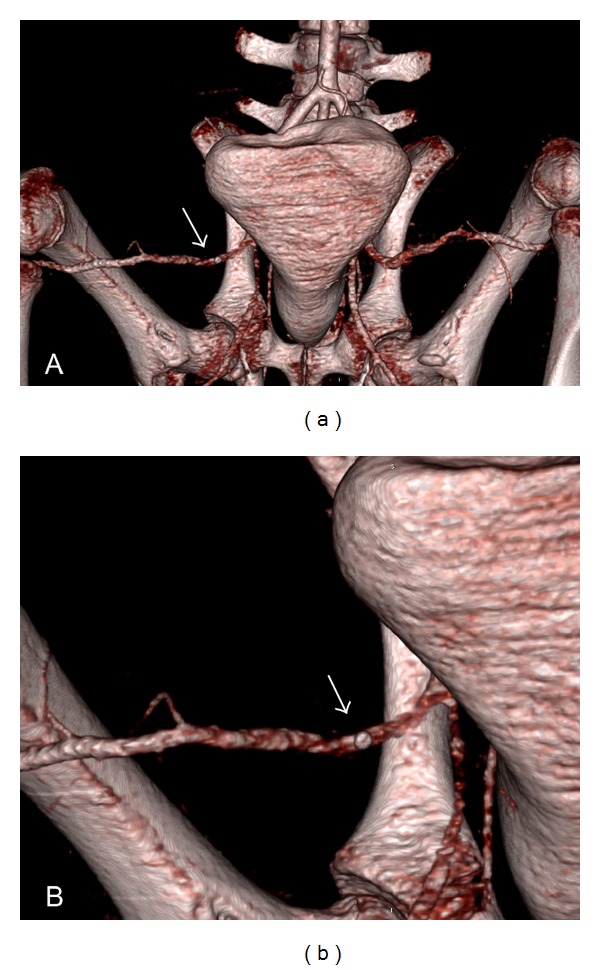
Three-dimensional visualization of a CT dataset in arterial contrast phase using volume rendering technique (VRT). (a) VRT after application of the VCD and (b) close-up of right FA with VCD clip in situ. Note unimpeded patency of the right femoral artery after VCD application. Note markedly different anatomy when compared to human vasculature.

All the aforementioned systems have significant benefits, such as reduction of time to haemostasis and early ambulation of patients and therefore less rates of secondary complications like deep vein thrombosis and pulmonary embolism, an increased patient comfort, and earlier discharge for some patients [4–14]. Nowadays, the use of VCDs causes no increase in access site complications compared to conventional manual compression [2, 3]. With VCD optimization and increasing experience of the physicians, a trend towards reduced local complications through the use of VCDs in comparison to manual compression can be observed [5, 7, 9, 15]. To date, no data on hemostasis after FA puncture using VCD in swine is available. Since the local anatomy of the vasculature in the pigs groin is different from the anatomy in humans (see also Figure 2), evaluation of the application and success of hemostasis are mandatory before routinely using VCD in swine. Moreover, due to the comparably deep localization of the FA in swine, a controlled compression of the FA against the femoral head is not possible.

In conclusion, hemostasis was successfully achieved in all animals while patency of the punctured FA was preserved. Thereby, no additional local compression was necessary.

## 5. Limitations

The presented study evaluates the safety of one particular VCD—the StarClose—Device. So, results cannot be transferred directly to other systems. Moreover, the study evaluated a rather small sample size when compared to studies evaluating VCD in humans, but, in our opinion, the sample size is sufficient for routine application of the system in animal trials.
